# Does Ivermectin Pollute the Surroundings of Swine Farms?

**DOI:** 10.1002/vms3.70634

**Published:** 2025-10-11

**Authors:** Alicia Maria Carrillo Heredero, Elena Butovskaya, Giulia Segato, Luigino Calzetta, Simonetta Menotta, Simone Bertini

**Affiliations:** ^1^ Department of Veterinary Sciences University of Parma Parma Italy; ^2^ Food and Feed Chemistry Department Istituto Zooprofilattico Sperimentale della Lombardia e dell'Emilia‐Romagna “Bruno Ubertini” (IZSLER) Brescia Italy; ^3^ Department of Medicine and Surgery University of Parma Parma Italy

**Keywords:** antiparasitic agents, ecotoxicology, environmental pollution, ivermectin, residues, swine

## Abstract

Pharmaceuticals are emerging environmental pollutants, with macrocyclic lactones like ivermectin raising concerns due to their widespread use and persistence. This study investigated ivermectin residues in intensive swine farms in Emilia‐Romagna, Italy, focusing on treated sows’ faeces and environmental samples. Two farms using ivermectin and a control farm without antiparasitic drugs were studied. Faecal, slurry, soil, and wastewater samples were collected at three treatment points: before, and 1 and 10 days post‐treatment. Ivermectin was administered either orally or by injection and analysed via liquid chromatography‐tandem mass spectrometry. Faecal residues varied significantly with administration route: orally treated sows had higher concentrations in faeces shortly after treatment (median = 930.25 µg kg^−1^) than injected sows (median = 14.84 µg kg^−1^). However, 10 days post‐treatment, injected sows exhibited higher residue levels in faeces, indicating different excretion patterns. Soil samples fertilised with farm slurry contained ivermectin residues, sometimes exceeding ecotoxicological thresholds for non‐target species like dung beetles and earthworms, with the highest concentration reaching 39.23 µg kg^−1^. No residues above detection limits were found in slurry or wastewater, likely due to ivermectin's hydrophobicity and dilution. The findings underscore the environmental risks of ivermectin use in swine farming and emphasise the need for sustainable drug management. Broader studies are necessary to refine residue distribution patterns and improve environmental guidelines, particularly for other livestock systems and soil types, to ensure ecological sustainability.

## Graphical abstract

This study assessed ivermectin residues in faeces and environmental samples from intensive swine farms in Emilia‐Romagna, Italy. Faecal concentrations varied by administration route, with oral treatment leading to higher early excretion. Residues in fertilised soil sometimes exceeded ecotoxic thresholds, highlighting environmental risks and the need for improved antiparasitic management in livestock systems.

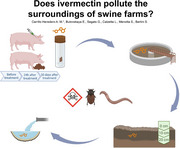

## 1 Introduction

Pharmaceuticals are a growing and now well‐established class of environmental pollutants with the potential to adversely affect ecosystems (Gildemeister et al. 2023; Moermond et al. 2023; Thornber et al. 2022). Their presence in the environment has become a matter of considerable scientific interest. Even at low concentrations, these substances can threaten human health by promoting the spread of resistance as well as by affecting both aquatic and terrestrial ecosystems (Bártíková et al. 2016; Boxall et al. 2004, 2022; Lertxundi et al. 2020). In response, the European Commission has proposed several measures to address the environmental release of human and veterinary pharmaceuticals (Directive 2008/105/EC 2008; Directive 2013/39/EU 2013). Macrocyclic lactones, a class of antiparasitic agents widely used in veterinary medicine, have been proven to pose a risk to the environment and to non‐target species living in soil, sediment, and water (Bai and Ogbourne 2016; Bártíková et al. 2016; Davies et al. 1997; Junco et al. 2021; Lumaret et al. 2012).

Veterinary drugs can enter the environment through slurry or manure from intensively farmed animals and through the dung or urine of grazing livestock. These substances can pollute surface waters via runoff, leaching, or drainage systems (Boxall et al. 2004; Kools et al. 2008; Li et al. 2013; Song and Guo 2014). Effluents from slurry and wastewater treatment facilities, along with direct defecation by grazing livestock, are major contributors to pharmaceutical pollution in aquatic ecosystems (Jaffrézic et al. 2017; Nikolaou et al. 2007; Obimakinde et al. 2017). Faeces of treated animals are one of the possible routes for the spreading of veterinary drug residues into the environment, and they may pose a threat to non‐target species (Boxall et al. 2004; Charuaud et al. 2019; Kelly et al. 2003). The biodegradability of drugs’ active principles differs, affecting how long they remain in the environment and their potential to accumulate in living organisms (Salvito et al. 2011). Antibiotics and parasiticides have been detected and reported in the environment by previous research, with high concentrations found in soils and surface waters near agricultural sites (Coleman et al. 2013; Delgado et al. 2023; Floate et al. 2004; Song et al. 2010; Song and Guo 2014).

Among macrocyclic lactones, several studies have identified ivermectin (IVM) as a potential environmental pollutant affecting animal as well as botanical species (Davies et al. 1997; Floate et al. 2004; Halley et al. 1989; Vokřál et al. 2019; Wall and Strong 1987). IVM has been authorised by the European Medicines Agency (EMA) and the American Food and Drug Administration (FDA) for use in both human and veterinary medicine (Drugs@FDA: FDA‐Approved Drugs; European Agency for the Evaluation of Medicinal Products & Committee for Veterinary Medicinal Products, 2009). It is available on the market in various forms, including feed premixes, spot‐on treatments, and injectable solutions (González Canga et al. 2009; Italian Health Ministry 2024). IVM is highly effective against a wide range of parasites and has an outstanding safety record when used according to the summary of product characteristics (SmPC). These attributes collectively contributed to its extensive adoption (Laing et al. 2017; Martin et al. 2021; Suvarna 2023). In swine farming, IVM is mainly used in breeding animals (sows and barrows) because of its long withdrawal period.

Although the Environmental Risk Assessment (ERA) of Pharmaceuticals did not classify IVM as a significant concern, several studies have highlighted the potential consequences of its widespread use. Previous papers have suggested possible accumulation of IVM in soil, emphasizing the necessity for cautious and sustainable use of this drug (Bai and Ogbourne 2016; Floate et al. 2004; Halley et al. 1989, 1993; Liebig et al. 2010; Mancini et al. 2020; Sanderson et al. 2007; Suvarna 2023; Vokřál et al. 2019, 2023). Parasite resistance has been confirmed by multiple studies, posing an additional challenge when its active residues persist in the environment without being degraded (Halley et al. 1993; Liebig et al. 2010). For these reasons, the extensive and systematic use of IVM in livestock has been questioned.

This field study aimed to assess IVM residues in intensive swine farms, from the treatment of swine through to slurry collection and distribution to soil and wastewater, in order to evaluate potential environmental contamination. The objective was to detect the presence of IVM, quantify it and compare these levels to potentially toxic concentrations for non‐target species in the environment.

## 2 Materials and Methods

**FIGURE 1:**
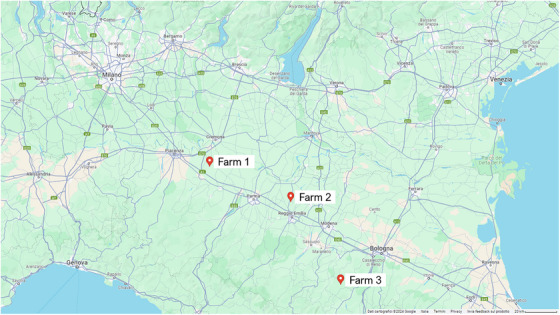
Map showing the location of farms involved in the study. They are all located in the Emilia‐Romagna region in Italy.

The criteria for farm inclusion were established to ensure adherence to the study design and the well‐being of the animals. Compliance with European and Italian animal health and welfare regulations, trained employees, and good husbandry practices were also required. Farms had to administer IVM for antiparasitic prophylaxis in sows routinely. All farms practiced intensive husbandry, housing sows in individual stables and providing a standard commercial diet formulated for sows. The Emilia‐Romagna region of Italy is known for its high concentration of livestock farms, especially pig farms. It has a thriving livestock industry and is home to large, well‐established companies specializing in producing heavy pigs, closely tied to the renowned Parma ham production. The swine breeding farms selected for the study were chosen to be representative of Italian swine production, as they were established businesses with significant animal output. Two farms in Emilia‐Romagna, Italy, were selected for this study because of the region's zootechnical vocation and the business industry's representativeness. The locations of these farms are N 44° 46′ 53.852″ E 10° 36′ 59.353″ for Farm 1 and N 44° 54′ 39.24″ E 9° 31′ 4.8″ for Farm 2, as shown in Figure 1. One additional farm (from now on referred to as Farm 3, N 44° 18′ 57.86″ E 11° 0′ 59.4″) that reported not using any antiparasitic drugs, including IVM, was chosen as a negative control. Its location is also shown in Figure 1.

Thirty sows for each type of treatment employed on each farm were randomly selected. Sampled sows received treatment according to SmPC: either injectable 300 mcg kg^−1^ of body weight (b.w.) in a single subcutaneous administration or oral IVM treatment 100 mcg kg^−1^ b.w. for seven consecutive days. Both products were Ivomec, purchased from Boehringer Ingelheim Animal Health Italia S.p.A. Faecal samples were obtained per rectum and placed into a 50 mL plastic sterile tube with a screw cap and stored at −20°C until analysis. Collections occurred at three time points: o ne hour before treatment (T0), one day post‐treatment (T1, in case of oral treatment, T1 was the eighth day after T0), and ten days post‐treatment (T10, eighteenth day after T0 in case of oral treatment). Farm 1 used injectable and oral treatment, while Farm 2 used only the injectable solution.

Slurry was sampled from the slurry collection and maturation tanks, which contained the slurry indicated by farmers to be used for field fertilisation. The sampling process involved thoroughly mixing the slurry to ensure a representative and homogeneous sample from the entire tank, and collecting the considered amount into a 50 mL plastic sterile tube with a screw cap and stored at −20°C until analysis. When multiple collection tanks were present on a farm, pooled samples were created. For each farm, ten 50 mL slurry samples were collected.

The slurry was spread on the fields in compliance with current regulations (Directive 91/676/CEE), which specifies the allowed spreading periods for each region based on climate conditions and air pollution levels. In the Emilia‐Romagna region, slurry spreading is allowed from October 1st to April 30th. Soil samples were taken from ten random points in the surrounding fields, where slurry was employed as fertiliser. Samples were taken at the surface and at depths of 10 and 20 cm. A shovel was used to dig the soil, which was cleaned and sanitised between each point. Soil was stored in a 50 mL plastic sterile tube with a screw cap and stored at −20°C until analysis. Drainage channels were identified close to the sampled fields, and wastewater was sampled with at least five samples for each farm. Wastewater was stored in a 50 mL plastic sterile tube with a screw cap and stored at −20°C until analysis.

IVM was quantified in samples through the LC‐MS/MS technique. The extraction and purification procedures were based on a previously published work by Carrillo Heredero et al. (2023), following adaptation to the matrices (faeces, soil, slurry, and wastewater) after validation according to Eurachem guidelines (Carrillo Heredero et al. 2024). The analysis was conducted for all matrices through liquid chromatography‐tandem mass spectrometry equipped with a Supelcosil C‐18 reverse phase chromatographic column (Supelco, USA) together with a Supelguard C‐18 reverse phase chromatographic precolumn (Supelco, USA). The method's detection limits (LODs) were verified during method validation and resulted in 0.66 µg kg^−1^ for stool, 0.54 µg kg^−1^ for soil, 0.36 µg kg^−1^ for slurry and 0.5 mg kg^−1^ in wastewater. The method's quantification limit (LOQ) was 1.5 µg kg^−1^ for all matrices. Blank and fortified (2.5 µg L^−1^) samples were employed for each pool of analysis conducted. The blank and fortified samples were subjected to the same extraction, purification, and analysis process as the real samples. The extraction and purification differed between matrices and were conducted as follows.

Briefly, 10 g of faecal sample was extracted with 50 mL of methanol. Faecal samples were purified first through activated neutral aluminium and then using solid phase extraction by means of C‐18 and Silica SPE columns. The extract was set to dry under nitrogen flow, re‐suspended in mobile phase and ultracentrifuged. The supernatant was placed in a vial with an insert and stored at −20°C until analysis.

A 10 mL volume of unfiltered, whole slurry sample was mechanically extracted through sonication and shaking, followed by ultracentrifugation. The extract was set to dry under nitrogen flow, resuspended in mobile phase and ultracentrifuged. The supernatant was placed in a vial with an insert and stored at −20°C until analysis.

Ten grams of soil sample were extracted with 50 mL of methanol. The extract was centrifuged, and the supernatant was set to dry under nitrogen flow, resuspended and ultracentrifuged. The supernatant was placed in a vial with an insert and stored at −20°C until analysis. Faecal samples were further purified, between extraction and drying, through activated neutral aluminium and solid phase extraction by means of C‐18 and Silica SPE columns. A 10 mL volume of slurry and wastewater samples was mechanically extracted through sonication and shaking, followed by ultracentrifugation.

A 10 mL volume of wastewater sample was mechanically extracted through sonication and shaking, followed by ultracentrifugation. The extract was set to dry under nitrogen flow, resuspended in mobile phase and ultracentrifuged. The supernatant was placed in a vial with an insert and stored at −20°C until analysis.

All the data were analysed using R v4.3.2 and RStudio. All packages were open source and included the following: Car, dplyr, factoextra, FactoMineR, ggbreak (Xu et al. 2021), ggplot2, ggpubr, readxl. Normality was evaluated among every single group and among the Farm, Time, Treatment, and Depth groups. Firstly, IVM concentrations were plotted through a density plot (ggdensity function of the ggplot2 R package). The Shapiro–Wilk normality test (shapiro.test in R stats package) was used to assess the distribution of all continuous variables. Descriptive statistics included mean and standard deviation for normally distributed data and median and range (minimum to maximum) for data that were not normally distributed. Statistical comparison between groups was tested through ANOVA and Tukey's HSD test (stats package in R), and the significance threshold was set at 0.05 (*), 0.01 (**), 0.001 (***).

## 3 Results and Discussion

Table 1 represents the total number of samples taken into consideration for the study.

**TABLE 1:** Consistency of samples collected by matrix, timepoint, treatment and depth.

Sample type		Farm 1		Farm 2	Farm 3	Total
Faecal	Time	Treatment				
	T0	Injection	30	30	30	120
		Oral	30			
	T1	Injection	30	30	30	120
		Oral	30			
	T10	Injection	30	30	30	120
		Oral	30			
Slurry			10	10	10	30
Soil	Depth					
	0 cm		10	10	10	30
	10 cm		10	10	10	30
	20 cm		10	10	10	30
Wastewater			5	5	5	15

The sows' parity ranged from first to sixth litters, and their ages ranged from 1 to 6. Overall, 360 faecal samples were collected, resulting in a total sample size of 120 sows. The normality of faecal samples was evaluated among farm groups, time groups and treatment groups. Then, the normality analysis was performed for every single group. No group showed a normal distribution of IVM concentration. A summary of descriptive statistics of faeces data is reported in Table 1S.

Faecal samples of animals that received injection treatment showed a median of 0.00 µg kg^−1^ (min = 0.00 µg kg^−1^; max = 319.88 µg kg^−1^) at T0, of 14.84 µg kg^−1^ (min = 0.00 µg kg^−1^; max = 117.58 µg kg^−1^) at T1, and of 98.20 µg kg^−1^ (min = 0.00 µg kg^−1^; max = 629.53 µg kg^−1^) at T10. The positivity rates, defined as the proportion of samples with results greater than 0.0 µg kg^−1^, for injection treatment were 21.67% (*n* = 13) at T0, 78.33% (*n* = 47) at T1, and 86.67% (*n* = 52) at T10. Faecal samples of animals that received the oral treatment showed a median of 0.00 µg kg^−1^ (min = 0.00 µg kg^−1^; max = 2.00 µg kg^−1^) at T0, of 930.25 µg kg^−1^ (min = 203.59 µg kg^−1^; max = 5767.44 µg kg^−1^) at T1, and of 11.16 µg kg^−1^ (min = 0.00 µg kg^−1^; max = 32.58 µg kg^−1^) at T10. The positivity rates for the oral treatment were 6.67% (*n* = 2) at T0, 100% (*n* = 30) at T1, and 96.67% (*n* = 29) at T10. The faecal samples from Farm 3 were confirmed not to have any residues of IVM at the three time points considered.

**FIGURE 2:**
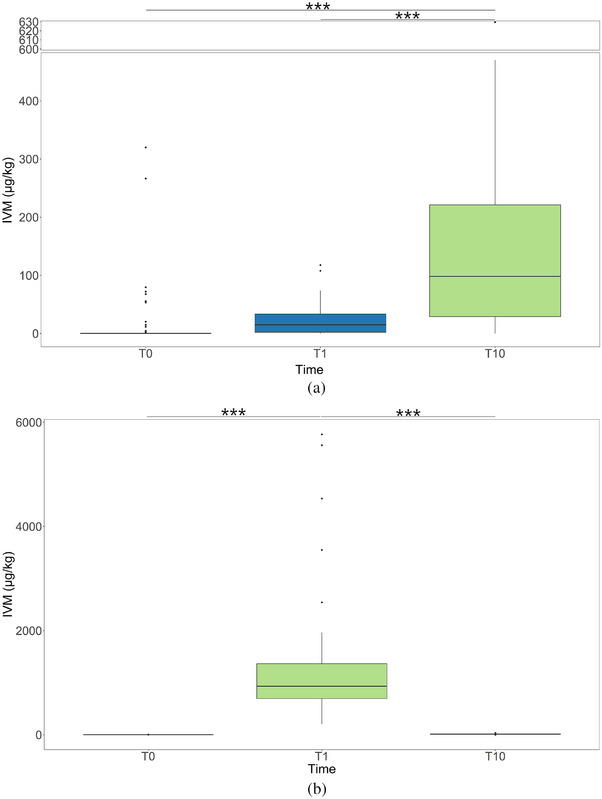
Results of faecal samples compared at the three time points: before treatment (T0), 24 h after treatment (T1), and 10 days after treatment (T10). Part (a) shows data relative to subcutaneously injected animals. Part (b) shows data of orally treated animals. Significance has been indicated as follows: ‘***’ = 0.001, ‘**’ = 0.01, ‘*’ = 0.05.

The highest median was obtained in the orally treated group at T1, while the lowest positive median was found for the same group at T10. The oral treatment regimen resulted in a notable and statistically significant difference in IVM levels between T0 and T1 (*p* < 0.001) and at T10 compared to T1 (*p* < 0.001). At the same time, no discernible variance was observed when comparing T0 to T10. Orally treated sows exhibited higher median IVM concentrations in faeces following treatment at T1 (930.25 µg kg^−1^) than injected sows (14.84 µg kg^−1^), as shown in Figure 2. Conversely, at T10, injected sows showed higher concentrations in faeces (98.20 µg kg^−1^) compared to orally treated sows (11.16 µg kg^−1^). On the other hand, injectable treatment didn't show a significant change between T0 and T1, while there was a significant increase when comparing T1 to T10 (*p* < 0.001) and between T0 and T10 (*p* < 0.001). Significant differences in IVM quantities were observed between the two treatments at all time points. Notably, the oral treatment produced a peak immediately after administration, while the regimen spans several days in the case of a single administration of injectable treatment. As suggested by previous work, this discrepancy may be attributed to the comparatively delayed distribution of IVM to the gastrointestinal tract associated with the injectable administration route (González Canga et al. 2009).

Pharmacokinetics of IVM through faeces have been investigated widely in cattle and a few other species, but not in swine (Cook et al. 1996; Fernandez et al. 2009; Gonzalez Canga et al. 2007; Herd et al. 1996; Laffont et al. 2001; Pérez et al. 2001). To our knowledge, no studies have focused on quantifying the environmental IVM residues produced by intensive swine farming. Pharmacokinetic studies in cattle show that IVM elimination in faeces reaches its highest concentration a few days after subcutaneous injection (3 days [Herd et al. 1996], 5.60 ± 3.44 days [Fernandez et al. 2009]). Fernandez et al. reported that the release of IVM in cattle faeces occurs over a period of more than a month, with only 35% of the total administered IVM being excreted within the first 30 days (Fernandez et al. 2009). On the other hand, oral administration of IVM in horses has shown a 90% clearance in 4 days, and IVM was detectable in faeces up to 40 days post‐treatment (Pérez et al. 2001).

Before treatment (T0), no statistically significant difference among treatments was detected. At T1, the difference in treatment was highly significant between oral and injection (*p* < 0.001) and between oral and the control group (*p* < 0.001). Ten days after treatment, the injection group differed significantly from both the control group (*p* < 0.001) and the orally treated group (*p* < 0.001). Canton et al. have underlined how the subcutaneous injection guarantees greater bioavailability while the oral treatment provokes a greater concentration of IVM in the faeces (Canton et al. 2018). Notably, several samples (*n* = 13) from the injection group were positive at T0 in both farms. The sows were not treated by mistake, and their management had been very rigorous. We hypothesise that other factors may have intervened in these cases. One study reported positivity in body fluids and tissue samples of untreated pigs living together with treated animals, and underlined the need for direct contact to result in positivity (Scott and McKellar 1992). We believe that in our study, animals had been positive since their last treatment, which may be due to slower clearance of the drug, which several groups have reported in the case of subcutaneous treatment (Fernandez et al. 2009; González Canga et al. 2009; Gonzalez Canga et al. 2007). The slower clearance could be affected by the animal's body condition scores, as IVM is proven to be highly lipophilic and could have accumulated in the fat tissue (Inoue et al. 2009; Suvarna 2023). Other authors have also underlined how the role of body fat may play a role that is still unclear in pigs (González Canga et al. 2009). To clarify these findings, further studies should correlate this data to body condition scores, back fat thickness and parity time‐lapse.

No analysed slurry samples had IVM concentrations above LOD and LOQ. We hypothesise that this is due to the aqueous nature of the slurry, which hinders IVM dilution and complicates analyte extraction. The absence of detectable IVM might also be attributed to its extreme dilution. The faeces are collected in tanks along with those of untreated animals. During the collection and cleaning process, substantial amounts of water are used to wash surfaces and floors, creating slurry and further diluting any traces of IVM.

**FIGURE 3:**
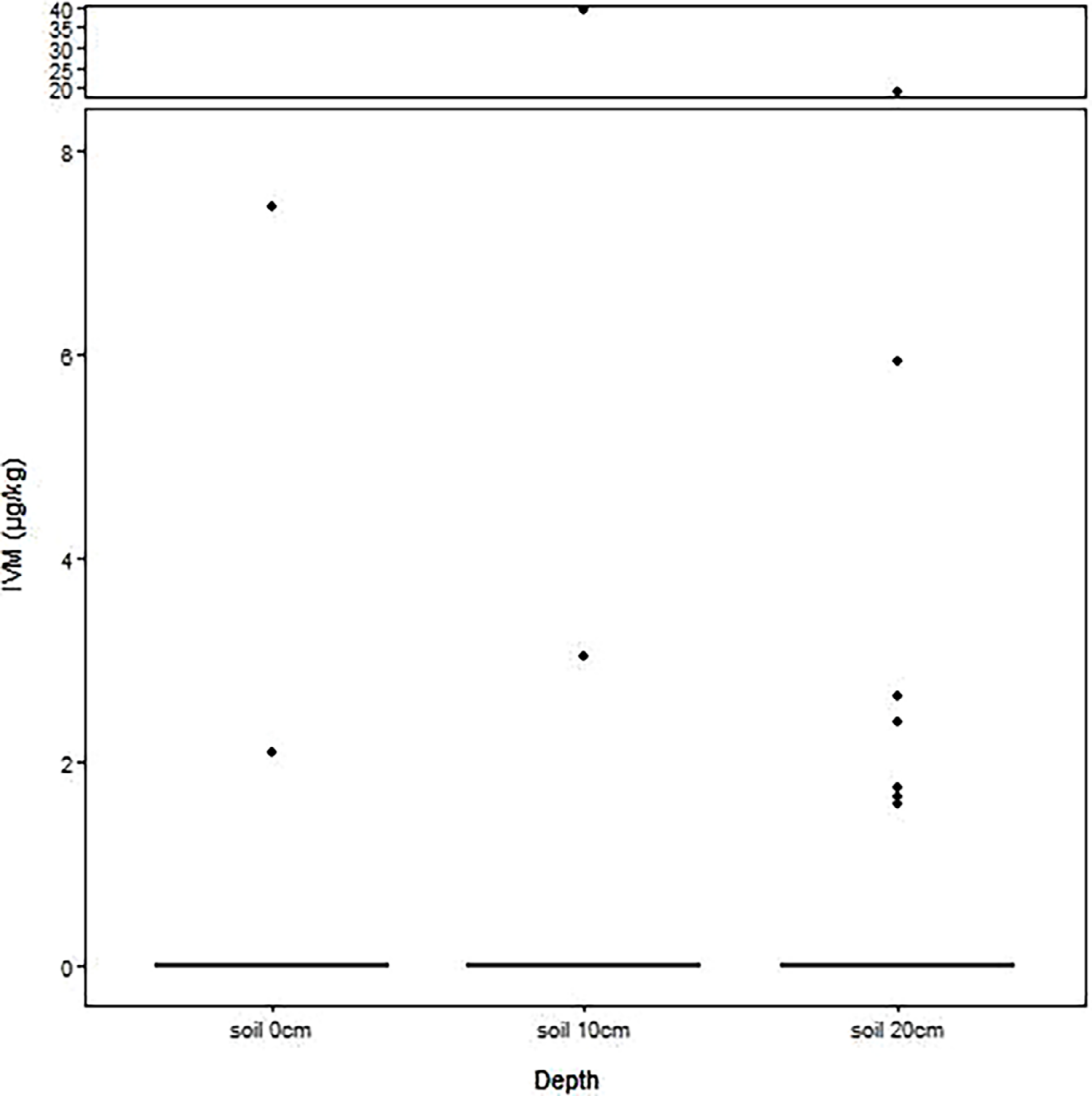
Results of soil samples compared by depth.

The normality of soil samples was evaluated among farm, time and depth groups. Then, the normality analysis was performed for every single group. None of the groups showed normal distribution. A summary of descriptive statistics of soil data is reported in Table 2S. Soil samples from Farm 1 showed a median of 0.00 µg kg^−1^ (min = 0.00 µg kg^−1^; max = 2.08 µg kg^−1^) at 0 cm of depth, of 0.00 µg kg^−1^ (min = 0.00 µg kg^−1^; max = 39.23 µg kg^−1^) at 10 cm of depth, and of 0.00 µg kg^−1^ (min = 0.00 µg kg^−1^; max = 19.2 µg kg^−1^) at 20 cm of depth. The positivity rates for Farm 1 were 10% (*n* = 1) at 0 cm of depth, 10% (*n* = 1) at 10 cm of depth, and 40% (*n* = 4) at 20 cm of depth. Soil samples from Farm 2 showed a median of 0.00 µg kg^−1^ (min = 0.00 µg kg^−1^; max = 7.44 µg kg^−1^) at 0 cm of depth, of 0.00 µg kg^−1^ (min = 0.00 µg kg^−1^; max = 3.04 µg kg^−1^) at 10 cm of depth and of 0.00 µg kg^−1^ (min = 0.00 µg kg^−1^; max = 5.94 µg kg^−1^) at 20 cm of depth. The positivity rates for Farm 2 were 10% (*n* = 1) at 0 cm of depth, 10% (*n* = 1) at 10 cm of depth and 30% (*n* = 3) at 20 cm of depth. No samples from Farm 3, which declared no use of antiparasitic drugs, were positive. Figure 3 shows no statistically significant difference among samples taken at different depths, and no significant difference was shown among farms at the same depth.

Indeed, the use of IVM in swine farms produces residues in the environment. While no statistically significant difference was found among different farms, this could be due to a relatively low number of samples or a limited number of farms included in the study. Although further sampling will be needed to confirm these results in a broader area, we can state that the highest concentration in the soil samples has been proven to be toxic for non‐target organisms. Indeed, the obtained value of 39.23 µg L^−1^ is greater than the concentration causing chronic toxicity in *Daphnia magna* (Di Paola et al. 2022) and disrupting behaviour and reproductive activity in dung beetles (González‐Tokman et al. 2017; Verdú et al. 2018). Such concentrations in soil are also higher than what has been proven to be safe for earthworms (33 mg kg^−1^) (Bai and Ogbourne 2016). An important consideration in evaluating macrocyclic lactones is their differing levels of toxicity to non‐target organisms. Research has shown notable variation among these compounds; for example, moxidectin is generally less harmful to invertebrates that inhabit dung than IVM. Specifically, dung fly mortality occurs at concentrations approximately ten times higher for moxidectin than for IVM, and IVM has been reported to be around five times more toxic to certain dung beetle larvae. These findings highlight the need to carefully evaluate the specific formulation of macrocyclic lactones when analysing their ecological effects in agricultural environments (Blanckenhorn et al. 2013; Jacobs and Scholtz 2015). Other compounds in this class, such as doramectin and eprinomectin, also pose environmental concerns due to their excretion in animal manure. Doramectin has been associated with reduced emergence of insects that breed in dung and is recognised as highly toxic to both dung‐associated fauna and aquatic species. Although eprinomectin tends to bind more strongly to organic matter in faeces—potentially reducing its immediate bioavailability—it still poses significant risks to dung and aquatic organisms, with added concerns about its persistence in soil and potential for sediment accumulation (de Souza and Guimarães 2022; Kolar et al. 2008).

No analysed wastewater samples had IVM concentrations above LOD and LOQ. As mentioned for slurry, we hypothesise that this could be due to the high hydrophobic nature of IVM, which tends to precipitate in water. Moreover, this matrix is subject to a possible dilution effect due to weather events occurring throughout the year.

This study faced a few limitations that impacted its scope and results. One of the primary challenges encountered was the extraction of IVM from water‐based samples, such as slurry and wastewater. The high water quantity in these samples probably caused the precipitation of IVM, which hindered the possibility of extracting it.

A larger sample size could provide more robust data and improve the statistical power of our findings. Although not negligible, the number of samples involved in our study was primarily influenced by logistical constraints during the Swine Fever epidemics in Italy, which caused a severe limitation in transporting materials of swine origin. The study necessitated multiple visits to each farm and the collection of substantial material, adding significant logistical challenges due to the disease outbreak. Indeed, the need for strict biosecurity measures and the reluctance of farmers to permit researchers' access partially hindered the sample collection. Furthermore, future perspectives will include more companies to enhance the representativeness of the data and provide a more comprehensive understanding of the studied phenomena in the swine district.

## 4 Conclusions

This study represents the first documented investigation to trace IVM residues in swine farming environments from faeces to soil, providing a novel perspective on the environmental fate of this widely used antiparasitic drug. These pioneering findings offer a crucial starting point for understanding how IVM may persist in the environment and potentially impact non‐target species.

Although no residues were detected in slurry and wastewater, the unexpected presence of IVM in soil suggests a previously unrecognised pathway for environmental accumulation, underscoring the need for further large‐scale and long‐term studies. Moreover, our research reveals substantial and previously unreported differences in environmental residue patterns between oral and injectable IVM administration routes, with implications for both residue persistence and excretion dynamics. Given IVM's known environmental resistance and toxicity to non‐target organisms, this distinction calls for reconsideration of administration practices in livestock to mitigate ecological risks. The study contributes to a One Health perspective, emphasizing the importance of integrating human, animal, and environmental health in the management of veterinary pharmaceuticals.

These findings highlight the need for thorough assessments to understand the environmental impact of antiparasitic drugs like IVM. Future research should build on this groundwork by extending residue monitoring to other animal species such as cattle and poultry, as well as to underexplored categories like boars and juvenile animals. Investigations into how IVM residues travel through different pathways—including manure management systems, runoff and soil infiltration—are essential. Further, assessing the potential for accumulation in various soil types and focusing on the organic components of slurry could provide crucial insights that were not accessible in this preliminary analysis.

By expanding this line of inquiry, we want to further delineate the ecological footprint of IVM and help develop evidence‐based guidelines for its sustainable use in animal husbandry. This study lays the groundwork for such efforts, offering a foundational model for future residue‐tracing research in agricultural ecosystems.

## Author Contributions

**Alicia Maria Carrillo Heredero**: conceptualisation, data curation, formal analysis, validation, writing – original draft. **Elena Butovskaya**: funding acquisition, project administration, writing – review and editing. **Giulia Segato**: formal analysis, funding acquisition, methodology, supervision, validation, writing – review and editing. **Luigino Calzetta**: supervision. **Simonetta Menotta**: funding acquisition, resources, supervision. **Simone Bertini**: conceptualisation, funding acquisition, resources, supervision, writing – review and editing.

## Ethics Statement

The University of Parma's Ethics Committee for Animal Experimentation (CESA) authorised the project with favorable ethical opinion number PROT. N. 14/CESA/2022.

## Conflicts of Interest

The authors declare no conflicts of interest.

## Supporting information




**Table S1**: Descriptive statistics of faeces data (µg kg^−1^).
**Table S2**: Descriptive statistics of soil data.

## Data Availability

All research data is available upon request to the corresponding author.
