# Supplementary material for: Does Ivermectin Pollute the Surroundings of Swine Farms?
Source: Vet Med Sci. 2025 Oct 11;11(6):e70634. doi: 10.1002/vms3.70634 (PMC12515052; doi:10.1002/vms3.70634)
Supplement: Supplementary file 1 — Table S1: Descriptive statistics of faeces data (µg kg−1). Table S2: Descriptive statistics of soil data. [file VMS3-11-e70634-s001.docx]

**Does ivermectin pollute the surroundings of swine farms?**

Table 1S - Descriptive statistics of feces data (μg kg^-1^).

| Treatment | Time | Median | Min. | Max. | Number of positive samples | Positive samples (%) | Total number of samples |
| --- | --- | --- | --- | --- | --- | --- | --- |
| Injection  (Farms 1 and 2) | T0 | 0.00 | 0.00 | 319.88 | 13.00 | 21.67 | 60 |
|  | T1 | 14.84 | 0.00 | 117.58 | 47.00 | 78.33 | 60 |
|  | T10 | 98.20 | 0.00 | 629.53 | 52.00 | 86.67 | 60 |
| Oral  (Farm 1) | T0 | 0.00 | 0.00 | 2.00 | 2.00 | 6.67 | 30 |
|  | T1 | 930.25 | 203.59 | 5767.44 | 30.00 | 100.00 | 30 |
|  | T10 | 11.16 | 0.00 | 32.58 | 29.00 | 96.67 | 30 |
| None  (Farm 3) | T0 | 0.00 | 0.00 | 0.00 | 0.00 | 0.00 | 30 |
|  | T1 | 0.00 | 0.00 | 0.00 | 0.00 | 0.00 | 30 |
|  | T10 | 0.00 | 0.00 | 0.00 | 0.00 | 0.00 | 30 |

Table 2S - Descriptive statistics of soil data.

| Farm | Depth | Median | Min | Max | Number of positive samples | Positive samples (%) | Total number of samples |
| --- | --- | --- | --- | --- | --- | --- | --- |
| Farm 1 | 0 cm | 0 | 0 | 2.08 | 1 | 10 | 10 |
|  | 10 cm | 0 | 0 | 39.23 | 1 | 10 | 10 |
|  | 20 cm | 0 | 0 | 19.20 | 4 | 40 | 10 |
| Farm 2 | 0 cm | 0 | 0 | 7.44 | 1 | 10 | 10 |
|  | 10 cm | 0 | 0 | 3.04 | 1 | 10 | 10 |
|  | 20 cm | 0 | 0 | 5.94 | 3 | 30 | 10 |
| Farm 3 | 0 cm | 0 | 0 | 0 | 0 | 0 | 10 |
|  | 10 cm | 0 | 0 | 0 | 0 | 0 | 10 |
|  | 20 cm | 0 | 0 | 0 | 0 | 0 | 10 |
